# QuKAN: A Quantum Circuit Born Machine Approach to Quantum Kolmogorov Arnold Networks

**DOI:** 10.1038/s41598-025-22705-9

**Published:** 2025-10-09

**Authors:** Yannick Werner, Akash Malemath, Mengxi Liu, Vitor Fortes Rey, Nikolaos Palaiodimopoulos, Paul Lukowicz, Maximilian Kiefer-Emmanouilidis

**Affiliations:** 1 Department of Computer Science and Research Initiative QC-AI, RPTU Kaiserslautern-Landau, Kaiserslautern, Germany; 2 Department of Physics, RPTU Kaiserslautern-Landau, Kaiserslautern, Germany; 3 Embedded Intelligence, German Research Center for Artificial Intelligence (DFKI), Kaiserslautern, Germany

**Keywords:** Quantum machine learning, Hybrid quantum models, Quantum KAN, Quantum physics, Qubits, Computer science

## Abstract

Kolmogorov Arnold Networks (KANs), built upon the Kolmogorov Arnold representation theorem (KAR), have demonstrated promising capabilities in expressing complex functions with fewer neurons. This is achieved by implementing learnable parameters on the edges instead of on the nodes, unlike traditional networks such as Multi-Layer Perceptrons (MLPs). However, KANs potential in quantum machine learning has not yet been well explored. In this work, we present an implementation of these KAN architectures in both hybrid and fully quantum forms using a Quantum Circuit Born Machine (QCBM). We adapt the KAN transfer using pre-trained residual functions, thereby exploiting the representational power of parametrized quantum circuits. In the hybrid model we combine classical KAN components with quantum subroutines, while the fully quantum version the entire architecture of the residual function is translated to a quantum model. We demonstrate the feasibility, interpretability and performance of the proposed Quantum KAN (QuKAN) architecture.

**Supplementary Information:**

The online version contains supplementary material available at 10.1038/s41598-025-22705-9.

## Introduction

As proposed by Liu et al.^1^, the generalization of the original Kolmogorov Arnold representation theorem^2^ to arbitrary width and depth can yield a model capable of holding its ground to classical Multi Layer Perceptron^3,4^ (MLP) in terms of interpretability and accuracy. The structure of the generalization of the original theorem into the KAN relies on trainable residual function that are represented as linear combinations of a set of basis functions. This strong resemblance between the linear combinations and the superposition representation of quantum mechanical wavefunctions motivates the implementation of the KAN as a quantum model. The potential benefits of a quantum approach to KAN go beyond structural analogy. Quantum systems inherently support the parallel evaluation of multiple functions via superposition, enabling operations on exponentially large Hilbert spaces^5,6^. Although, previous work exist on quantum KANs, it is largely preliminary. Thus, QKAN^7^ is limited to translating the residual function as a unitary representation and has not yet been evaluated on its training performance. An initial concept for a Variational Quantum KAN (VQKAN) has been presented in^8–10^. However, despite being more robust against noise, the enhanced version^10^, where the residual functions are evaluated through the construction of a tiled matrix using sum operators, introduces an exponential overhead when the number of layers is increased.

In this work, we propose a feasible and simple quantum generalization of KAN via Quantum Circuit Born Machines^11^ (QCBM). These models learn to generate target probability functions using the probability interpretation of quantum physics given by the Quantum Born rule^12^. In this paper we go beyond probabilistic sampling (which is how QCBMs are usually used) and propose encoding entire residual functions into quantum states via weighted superpositions. Using projective measurements we can evaluate multiple functions at once allowing us to represent classical KAN residual functions as a trainable quantum circuit by including a division of the computational basis into labelling and position. We then propose an even more general form of the network that includes superposition interpretation of all the parts of the residual functions, yielding an effective fully quantum residual.

Finally, we demonstrate the models capabilities and performance on simple datasets with Binary Classification and function approximation and compare to sparsely available results from an Enhanced VQKAN^10^, and to a trainable version of QKAN^7^. Furthermore, we also present results from comparable Variational Quantum Circuits, a rigid grid pyKAN and two- and four layered MLP where we evaluate the *make_moons* and Iris^13^ dataset.

## Methods

In this section, we present the implementation of the Hybrid Quantum KAN. Our model leverages the structural resemblance between the definition of B-Splines^14^ as linear combinations of functions and the general representation of an arbitrary quantum state as a linear combination of basis states. We provide a brief overview of the QCBM, the superposition-based function approximation, the readout mechanism, and the construction of both single and combined QuKAN residual functions.

### Quantum Circuit Born Machines

Quantum Circuit Born Machines are a promising tool in the regime of unsupervised generative learning of quantum circuits due to their high expressive power^15^. The training utilizes the probabilistic interpretation of the wave function of a quantum state in a given representation, as described by the Quantum Born rule^12,16^. It states that the probability of measuring a quantum system in a particular state is equal to the squared magnitude of the amplitude resulting from the projection of the wavefunction onto that state. This stands in contrast to the idea of Boltzmann machines that leverage thermal distributions^17^. Given a target function as a dataset of independent samples, the QCBM uses projective measurements in the computational qubit basis. The outcome probability distribution is then expected to resemble the discretized target data. For our purposes, the quantum circuit always starts in the state $|0\rangle ^{\bigotimes n}$, representing the $n-$qubit computational basis state $|00\dots 00\rangle$. It consists of strongly entangling layers^18^, composed of parametrized rotation gates and nearest neighbour controlled NOT gates. The loss is computed from the measured output, and the circuit parameters are updated using gradient based learning^11^.

**Fig. 1:**
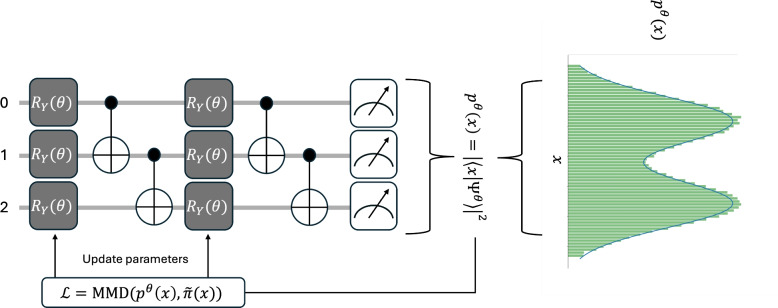
Sketch of the Quantum Circuit Born Machine learning algorithm: Starting from the state $|0\rangle ^{\bigotimes n}$ we process through a quantum circuit containing strongly entangling layers, so parametrized rotations as well as CNOT gates. The full computational basis is measured via projective measurements and the squared maximum mean discrepancy (MMD) loss is calculated. Note that the comparison here goes along the normalized target distribution $\tilde{\pi }(x)$, since the total probability $p^{\theta }(x)$ across the computational basis is constrained to sum to one. After training we can perform another run and generate the target.

In order to work in low dimensional feature spaces we map the data using Kernel methods as proposed by Gretton et al^19^. As the QCBM uses projective measurements we want to highlight that the input features are given only at the final stage, defined by the position indicated by the binary string obtained from the readout. This resembles the probability amplitude of the corresponding computational basis state, see Fig. 1. In the following we will use the QCBM as a form of amplitude embedding for the corresponding spline basis functions. One can immediately use Mottonen encoding^20^, which introduces a large overhead due to its exponential circuit depth scaling with the amount of qubits.

### Superposition distribution learning

For later purposes we want to generalize the learning capabilities of a QCBM using the quantum superposition principle. This allows a quantum system to represent multiple classical target functions simultaneously in contrast to conventional models. Specifically, we show that a single quantum state can encode a collection of discretized probability distributions, each corresponding to a different target, within its structure. To this aim we introduce a division of the computational basis of the circuit into labelling qubits (denoted by *i*) and position qubits (denoted by *k*). The total Hilbert space in this case is given by the tensor or Kronecker product^21^ of the labelling and position subspaces $H=H_{label}\bigotimes H_{pos}$, and the corresponding overall state can be written as

1 $$\begin{aligned} {|{\Psi }\rangle } = \sum _{i=0}^{N_{L}-1}c_i {|{i}\rangle }\otimes {|{\psi _i}\rangle } = \sum _{i=0}^{N_{L}-1}c_{i}\sum _{k=0}^{N_{P}-1} d_{ik}{|{i,k}\rangle }, \end{aligned}$$

where $\sum _{i}|{c_{i}}|^2=1$, and ${|{\psi _{i}}\rangle }=\sum _{k}d_{ik}{|{k}\rangle }$ denotes the normalized position state with $\sum _{k}|{d_{ik}}|^2=1$. We define ${|{i,k}\rangle }={|{i}\rangle }\otimes {|{k}\rangle }$. The coefficients $c_{i}$’s are initialized uniformly as $c_{i}=1/\sqrt{N_L}$ for all *i*. Here the index *i* ranges over the $N_{L}$ labelled states, while *k* runs over the $N_{P}$ position states.

**Fig. 2:**
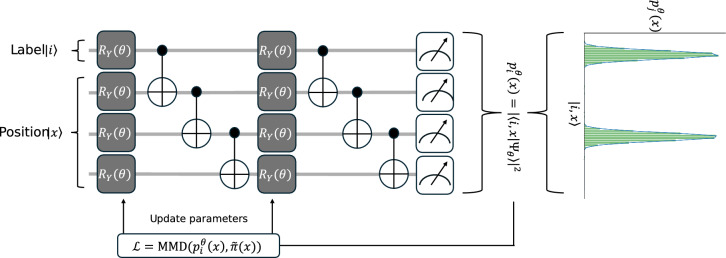
Sketch of the training of the QCBM for parallel superposition learning of two target functions. The process follows the same algorithm as shown in Fig. 1. The difference lies in the comparison for the optimization. Here we compare to the state representing both functions as given in the Kronecker basis denoted by $\tilde{\pi }(x)$.

To obtain a particular target probability distribution $\tilde{\pi }(x)$ at a given input feature point *x*, we perform a projective measurement^5^ in the computational basis over the combined label and position qubits. The outcome probability of measuring a particular basis state is given by

2 $$\begin{aligned} p^\theta _j(x) = |\langle j,x| \Psi ^{\theta }\rangle |^2 =|{\sum _{i} c_{i}\langle j|i \rangle \langle x | \psi ^{\theta }_{i}\rangle }|^2=|\sum _i c_{i} \delta _{ij}\langle x| \psi _{i}^{\theta }\rangle |^2 =|{c_{j}\langle x| \psi _{j}^{\theta }\rangle }|^2=|c_{j}|^2|{d^{\theta }_{j}(x)}|^2 \end{aligned}$$

where $\theta$’s are computed in the pre-training phase and $d^{\theta }_{j}(x)=\langle x|\psi _j^{\theta }\rangle$. Note that taking the absolute squared of the complex valued amplitudes maps them to real valued probabilities. The $\delta _{i,j}$ denotes the Kronecker delta that represents the orthonormality of the computational basis on which we encode. The effect of this projection is to isolate the squared amplitude which, guaranteed by the Born rule^12,16^, gives the probability of measuring a specific label and position pair. Crucially, this approach allows the QCBM to be trained just as the original formulation by adjusting the the weights of the parametrized unitaries such that the measurement matches the empirical data, see Fig. 2. The key difference lies in the encoding that utilizes quantum benefits, namely superposition.

### Hybrid KAN Residual Functions

In this section we are going to introduce a hybrid formulation of the Quantum KAN residual function utilizing the superposition distribution learning described in the previous section. As this directly maps onto the classical KAN architecture, we will first provide a summary of the classical counterpart.

#### Summary of classical KAN

The Kolmogorov–Arnold representation theorem is a foundational result in multivariate function theory, stating that any continuous function of multiple variables can be represented as a superposition of continuous univariate functions and addition. Formally, the theorem states that for any continuous multivariate function on a bounded domain $f: [0,1]^n \rightarrow \mathbb {R}$, there exist continuous functions $\phi _i$ with a single variable and $g_j$ such that:

3 $$\begin{aligned} f(x_1, \dots , x_n) = \sum _{j=1}^{2n+1} g_j\left( \sum _{i=1}^{n} \phi _i(x_i)\right) \end{aligned}$$

This result, originally proved by Kolmogorov and later refined by Arnold^22,23^, has profound implications in the field of approximation theory. It guarantees that multivariate continuous functions can be constructed using only univariate function compositions and additions, without requiring explicit multivariate non-linearities.

KANs^1^ build upon Kolmogorov–Arnold representation theorem. While there are only two-layer non-linearities and a small number of terms $(2n+1)$ in the hidden layer according to this theorem, the authors generalized the network to arbitrary widths and depths by defining a single KAN layer $\phi (x)$ in the following way: since the function $\phi _i(x_i)$ in Eq. (3) is a univariate function, it can be parametrized as a B-spline curve *f*(*x*) given by

4 $$\begin{aligned} f(x) = \sum _{i}\tilde{c}_i B_i(x), \end{aligned}$$

with learnable coefficients $\tilde{c}_i$ of local B-spline basis functions $B_i(x)$ as shown in Eq. (4), where $\tilde{c}_i$ is the trainable parameters. Theoretically, *f*(*x*) can be implemented using the KAN layer $\phi _i(x_i)$. A residual architecture was designed to enhance its optimization. Consequently, the KAN layer was defined as

5 $$\begin{aligned} \phi (x) = w_b \text {SiLU}(x) + w_s \sum _i \tilde{c}_i B_i(x), \quad \textrm{with} \quad \text {SiLU}(x) = \frac{x}{1+\text {e}^{-x}}, \end{aligned}$$

where $w_b$ and $w_s$ are trainable weights, retained in the original implementation to control the overall magnitude, and SiLU is the Sigmoid-weighted Linear Unit.

For a machine learning task the goal is for every input *x* to find a mapping $\phi (x)$ that closely approximates the true mapping $\phi ^*(x)$. In the KAN architecture, the process begins with an initial approximation defined by a basis activation $b(x):=\text {SiLU}(x)$. Following Liu et al^1^, the residual *r*(*x*) is then defined by the difference between the current and the optimal representation $r(x) = \phi ^*(x) - b(x)$ or rearranging $\phi ^*(x) = r(x) + b(x)$. The goal for KAN is to approximate the residual by using splines with learnable weights, i.e. $r(x):= f(x)$. Consequently, the overall transformation in a KAN layer is expressed as a composition and sum of residual functions added to the chosen basis function, as formalized in Eq. (5).

In traditional neural networks such as MLPs, each layer computes affine transformations followed by fixed element-wise non-linearities (e.g., ReLU or tanh). While these architectures are known to be universal approximators under certain conditions, they often require large numbers of neurons or layers to approximate complex functions effectively. However, KAN replaces the fixed scalar weights between neurons with learnable univariate functions (B-splines). Instead of each edge carrying a scalar weight, it carries a learnable function $\phi (x)$, enabling the network to directly approximate the decomposition described in Eq. 3. This allows KANs to express more complex functions with fewer neurons and deeper theoretical grounding. We want to emphasize here that the definition of the B-spline part in the residual function already has great resemblance to the superposition structure of a quantum mechanical wavefunction. The goal of the next section is to see now how we can combine the QCBM representation of a wavefunction in position space to the learning scheme of a classical KAN residual function.

#### Quantum representation of the Residual Functions

With the proposition of superposition based distribution learning via QCBM methods, we have demonstrated the possibility of encoding multiple classical functions into the probability distribution generated by the measurement statistics of a state. Building upon the previously introduced label-position register decomposition in the computational basis, we now apply this framework to train the network on a predefined set of discretized B-spline basis functions given by the Cox-de-Boor recursion^14^. These functions denoted by $B_i(x)$ form the building block of the classical KAN residual function which is given by Eq. (4).

**Fig. 3:**
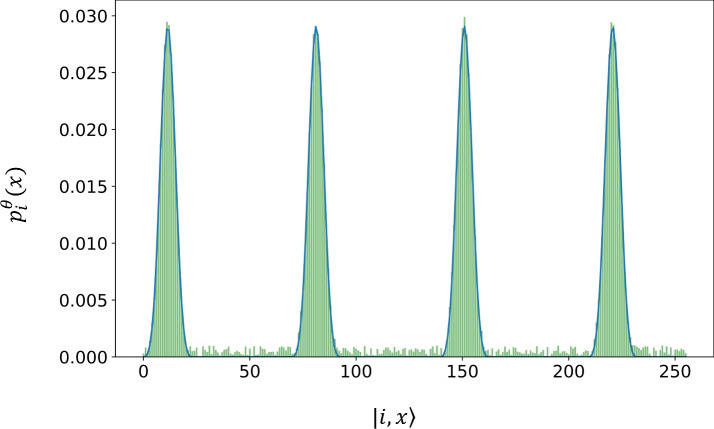
QCBM probability output for normalized B-spline basis functions on discretized input interval. In this example, the number of labelled states is $N_L=4$, corresponding to two label qubits, while the number of position states is $N_P=64$, corresponding to six qubits of the position register.

**Fig. 4:**
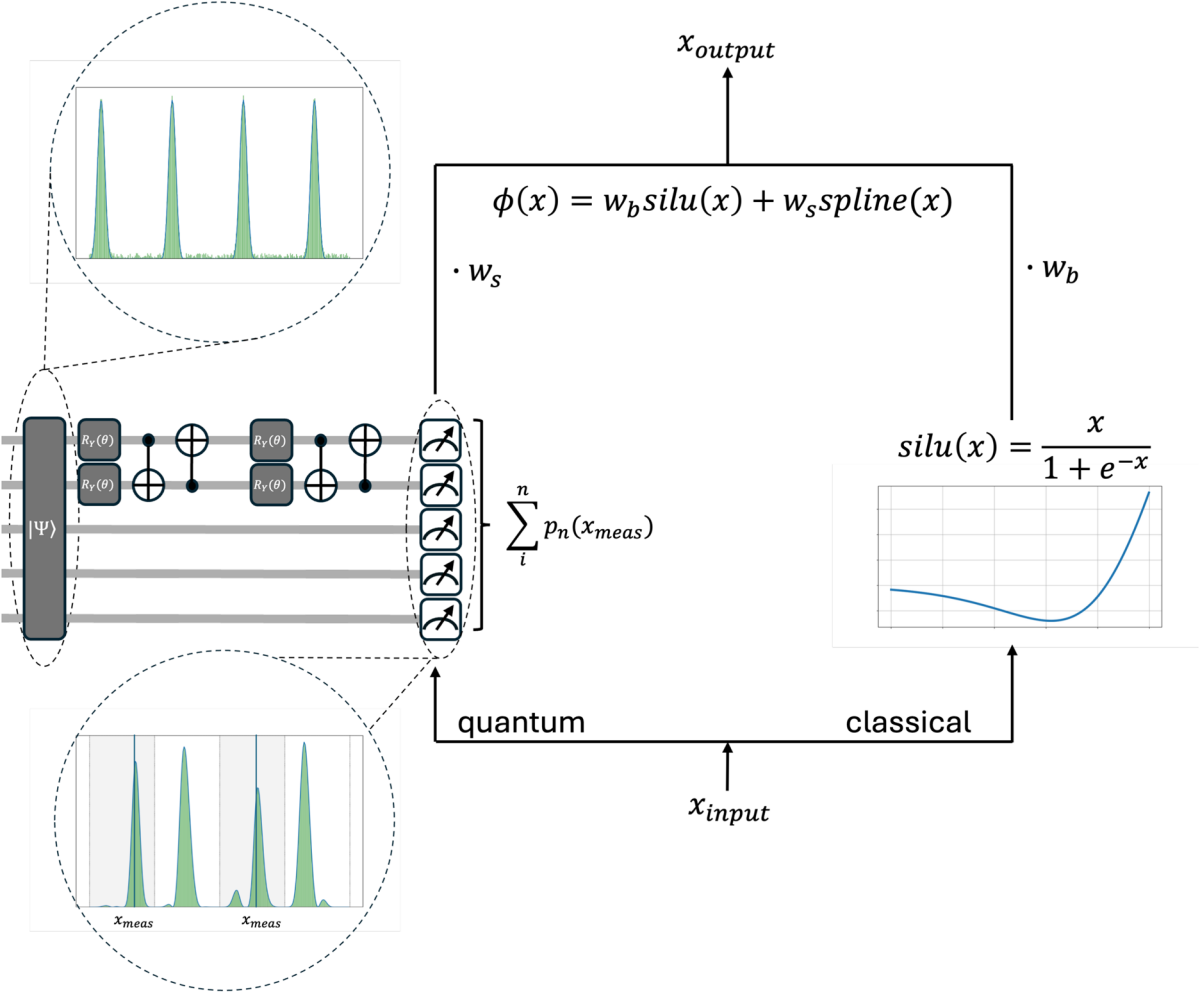
Architecture of a single Hybrid QuKAN residual function. The input data gets processed through a classical (the SiLU function) and a quantum transfer. The quantum transfer is based in the pre-trained QCBM Spline function encoding and optimizes the prefactors of the linear combination. Both get weighted and summed up to produce the output.

In the quantum formulation we encode the evaluation of the basis functions into the (normalized) amplitudes of a quantum state.

6 $$\begin{aligned} {|{f_{\text {init}}}\rangle }=\sum _{i=0}^{N_{L-1}} c_{i}|i\rangle |\beta _i\rangle ,\quad \textrm{and} \quad f_i(x)={\langle {i,x}|}{|{f}\rangle }, \quad \beta _i(x)={\langle {x}|}{|{\beta _i}\rangle } \end{aligned}$$

where *i* indicates the label, *x* denotes the position in the corresponding qubit register and $c_{i}$ represents the amplitudes of the $\beta _i$ states which are pre-trained by the QCBM, see Eq. (2). As mentioned earlier, the amplitudes are initially set uniformly as $c_{i}=1/\sqrt{N_L}$. An example of the corresponding probability distribution for the initial state ${|{f_{\text {init}}}\rangle }$ is shown in Fig. 3. The state is subsequently evolved by a quantum circuit of the type shown in Fig. 4, in which the parametrized (trainable) gates are restricted to the labelling register. Thus, the evolution can be expressed as

7 $$\begin{aligned} {|{f}\rangle }=(\hat{U}_L(\theta )\otimes \hat{I}_{P}) {|{f_{\text {init}}}\rangle }, \end{aligned}$$

where $\hat{U}_L(\theta )$ is composed by the label-qubit rotations and CNOTs, and the $I_P$ is the identity on the position register. The outcome probability distribution is given by

8 $$\begin{aligned} p_f(x)&= \sum _{j\in S} |{\langle {j,x}|} (\hat{U}_L(\theta )\otimes \hat{I}_{P}) {|{f_{\text {init}}}\rangle }|^2= \sum _{j\in S} | {\langle {j}|} \hat{U}_L(\theta ) \sum _{i=0}^{N_L-1} c_i \beta _i(x){|{i}\rangle }|^2 = \sum _{j\in S} |\sum _{i=0}^{N_L-1} c_i U_{ji}(\theta ) \beta _i(x)|^2, \end{aligned}$$

where $U_{ij}(\theta )={\langle {j}|} \hat{U}_{L}(\theta ) {|{i}\rangle }$ and by *S* we denote a partition of $j \in \{0,1,2,...,N_{L}-1 \}$. The aim of training the labelling register is to adjust how much each basis function is weighted at the output of the circuit. To this end, it is important to understand that if the first sum of Eq. (8) runs over all *j*’s then $p_{f}(x)$ becomes independent of $\theta$, rendering the circuit training impossible. This is shown analytically at the Supplementary Material. To overcome this limitation, one may either restrict the summation to a subset of *j* values or extend the trainable part of the circuit to the position register. In this work, for Eq. (8) we take $j=0,2,4,...,N_L-1$. We have to note however, that for the datasets considered in this work, the precise choice of partition is not critical, provided that the summation does not encompass all values of *j*. More generally, we expect that the required number of splines (*j* values) depends on the complexity of the dataset, with a minimum number necessary to ensure efficient circuit training.

To preserve the correct magnitude of the function approximation, we recognize that a normalization factor introduces downscaling of the amplitudes due to the probabilistic nature of quantum states^21^. However, since the normalization scaling is a constant factor, we can correct it either during post-processing or by absorbing it into the training objective. In particular, the classical coefficients can be reconstructed as

9 $$\begin{aligned} f(x)\sim p_f(x) , \textrm{when}\quad \tilde{c_i} \sim |c_i U_{ji}(\theta )|^2,\quad \textrm{and} \quad B_i(x) = |\beta _i(x)|^2 \end{aligned}$$

ensuring that the learned distribution matches the target functions amplitude structure after rescaling.

#### Hybrid QuKAN Residual Function

To understand how information propagates through the QuKAN architecture, we begin by analysing the processing of a single hybrid residual function. This unit combines a Quantum Function Evaluator (QFE), trained to approximate a set of pre-trained basis functions, with a classical non-linear transformation. This is analogous to the architecture of the classical KAN. The sequence of steps involved in implementing a single hybrid residual function is schematically depicted in Fig. 4.

The first step is to discretize the input data. This step is necessary due to the finite resolution of quantum registers, which restricts the number of distinguishable input values encoded into the position qubits by the exponentially large Hilbert space. Let $n_x$ denote the number of available qubits of the position register. Then the input feature space is partitioned into $2^{n_x}$ equally distanced points between the minimum and the maximum of the input range. Formally, if *X* is the set of input features, then

10 $$\begin{aligned} X = \{x_0,x_1,...,x_{2^{n_x}-1}\}, \,\,x_i = \min (X) + i\Delta x, \,\,\,\,\Delta x = \frac{\max (X)-\min (X)}{2^{n_x}-1}. \end{aligned}$$

For any given input feature we determine the nearest discretized point by

11 $$\begin{aligned} x_{\text {meas}} = \text {argmin}_{x\in X}|x-x_{\text {input}}|, \end{aligned}$$

and assign it to that position in our position register which determines where we are going to perform the projective measurement for the readout.

In the next step, we initialize our quantum circuit with a QCBM pre-trained quantum state representing a set of evaluations of B-spline basis functions as described in the previous section. The initial state is given as an equal superposition over the labelling register and an example of this state is highlighted in Fig. 4 by the dashed circle above the quantum circuit. During forward propagation the labelling qubits that index and weight the basis functions are passed through multiple parametrized entangling layers, analogous to classical weight training KANs. These trainable gates are set to optimize the coefficients in the linear combination of the basis functions, tailored to the given task. The position qubit register remains fixed and only comes into play by determining the position of the projective measurement. This measurement projects the total state onto the pre-determined $x_{\text {meas}}$ according to Eq. (8). A schematic example of the measurement process is shown in Fig. 4, highlighted by the dashed lines below the quantum circuit. The splines contributing to the summation in Eq. (8) are indicated by the gray background (corresponding to $j=0,2$), while the black vertical lines mark the position of $x_{\text {meas}}$. Optionally, now the obtained probability can be upscaled again.

In parallel to that, the unpreprocessed input is processed through a classical non-linear activation, such as (in analogy to the classical KAN) the SiLU. The final output of the residual function is obtained by summing over the quantum and classical part with the introduction of trainable scaling parameters as

12 $$\begin{aligned} f_{\text {Residual}}= w_f p_f(x) + w_s \text {SiLU}(x). \end{aligned}$$

With the definition of the structure of a single QuKAN residual function, we can extend this construction to form a full network. The architecture mirrors the structural paradigm of the KAR as introduced in the generalized KAN^1^, combining residuals into feed-forward networks of arbitrary width and depth.

### Full Quantum KAN

**Fig. 5:**
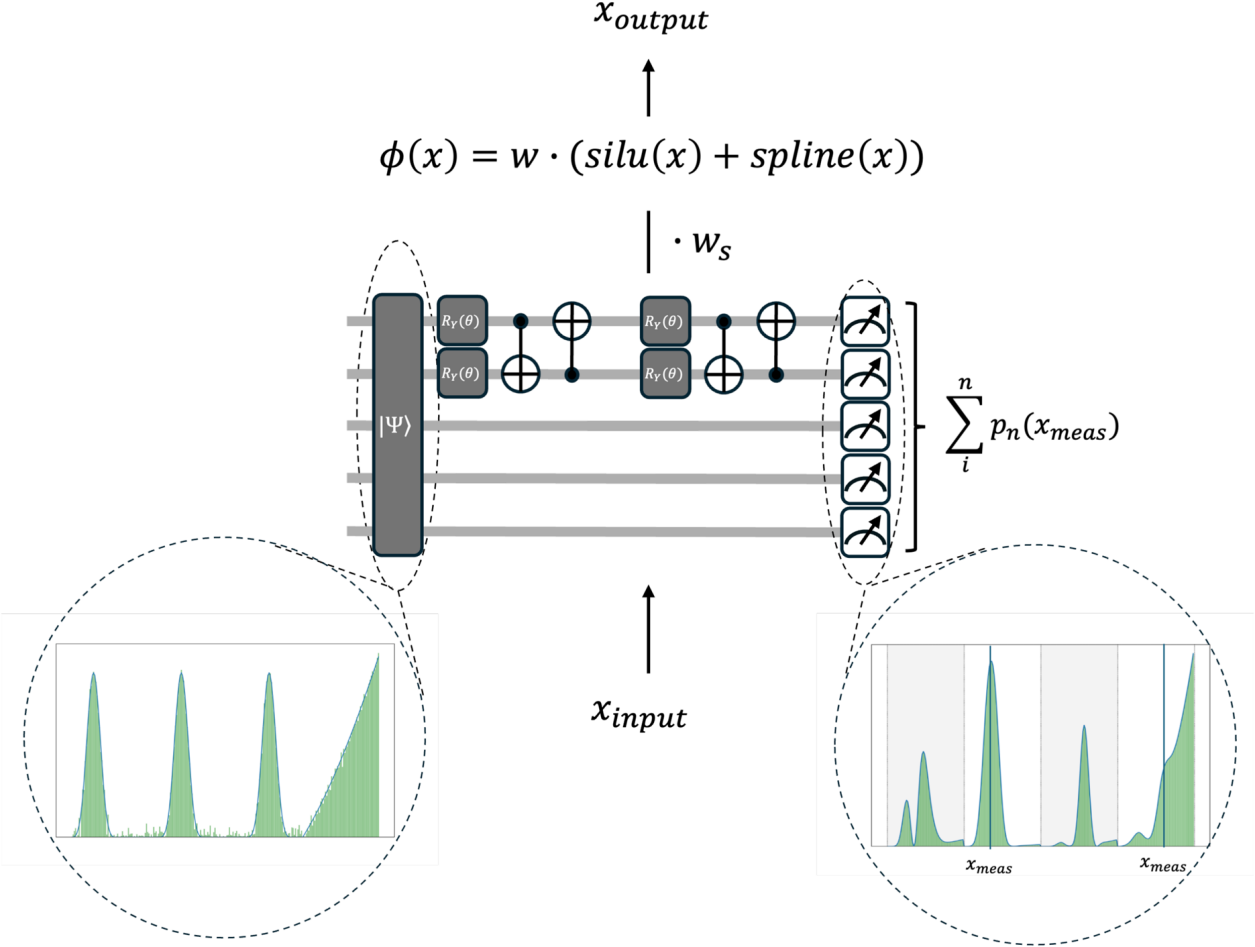
Architecture of a single FQuKAN residual function. Contrary to the hybrid version (see Fig. 4) the input is only processed through a quantum transfer. The quantum transfer is based in the pre-trained QCBM that now integrates the Splines as well as the SiLU, optimizing the prefactors of the linear combination. The output value is weighted again and transferred to the next layer.

In this section, we present the implementation of a full Quantum KAN (FQuKAN). The main idea is to absorb the $\operatorname {SiLU}$ activation into the spline superposition, which is subsequently normalized and encoded as a quantum state:

13 $$\begin{aligned} \phi (x) = w_b\,\operatorname {SiLU}(x) + w_s \sum _{i=1}^{N_S} \tilde{c}_i\,B_i(x) = \sum _{i=1}^{N_S+1} a_i\,\psi _i(x), \end{aligned}$$

where

$$\begin{aligned} \psi _i(x)= {\left\{ \begin{array}{ll} B_i(x), & i=1,\dots ,N_S\\ \operatorname {SiLU}(x), & i=N_S+1 \end{array}\right. } \qquad a_i= {\left\{ \begin{array}{ll} w_s\,\tilde{c}_i, & i=1,\dots ,N_S\\ w_b, & i=N_S+1, \end{array}\right. } \end{aligned}$$

and where $N_S$ denotes the number of splines. The fully quantum residual function that consists of a weighted superposition of spline basis functions as well as the SiLU. This changes the form of the hybrid residual function such that the classical part is absorbed into the quantum side. We evaluate in parallel not only the splines at a given input feature using projective measurement but also include the SiLU via pre-training a QCBM on the complete superposition of the classical KANs residual function (see Fig. 5). It is possible to retain both the normalization factor and a shift, derived from the SiLU, as numerical constants used to rescale the function during the encoding process. Specifically, the function is shifted upward by its minimum value within the corresponding interval to eliminate negative values, and the magnitude of this shift is stored. After propagation through the quantum circuit and measurement, the resulting probability distribution is rescaled and shifted back using the stored normalization factor and shift value. While the normalization can be applied uniformly to the entire encoded state, the shift must be applied exclusively to those probabilities corresponding to functions that were preprocessed with a shift.

### Summary of the Methods

In this section, we have introduced the architecture and core mechanism of the QuKAN as a hybrid model that integrates quantum-enhanced function representation. By leveraging the superposition principle we have demonstrated how a single quantum state trained via a QCBM can encode multiple discretized B-Spline basis functions simultaneously. This followed a decomposition of the computational register into labelling and position qubits allowing us to evaluate multiple functions with projective measurements of the position register. This hybrid architecture maintains the functional interpretability and compositional power of classical KANs, while introducing quantum native parallelism and probabilistic expressibility.

## Results

In this section we will present a few benchmarks of the performance of the hybrid quantum KAN in both classification and function approximation tasks. Regarding classification tasks we evaluated our method in two datasets, namely the two moons dataset as provided in the scikit-learn^24^ *make_moons* function and the Iris^13^ dataset. For function approximation, we tested our approach with two variable functions, including some evaluated in Wakaura et al.^10^.

### Binary Classification

For the classification tasks we will compare our model’s performance to that of a Variational Quantum Classifier (VQC) using different encoding strategies. Since the Quantum KAN architecture is close to a VQC with pre-training and ancillas, we also show that the pre-training, namely the encoding of the spline basis functions, has a positive effect.

**Fig. 6:**
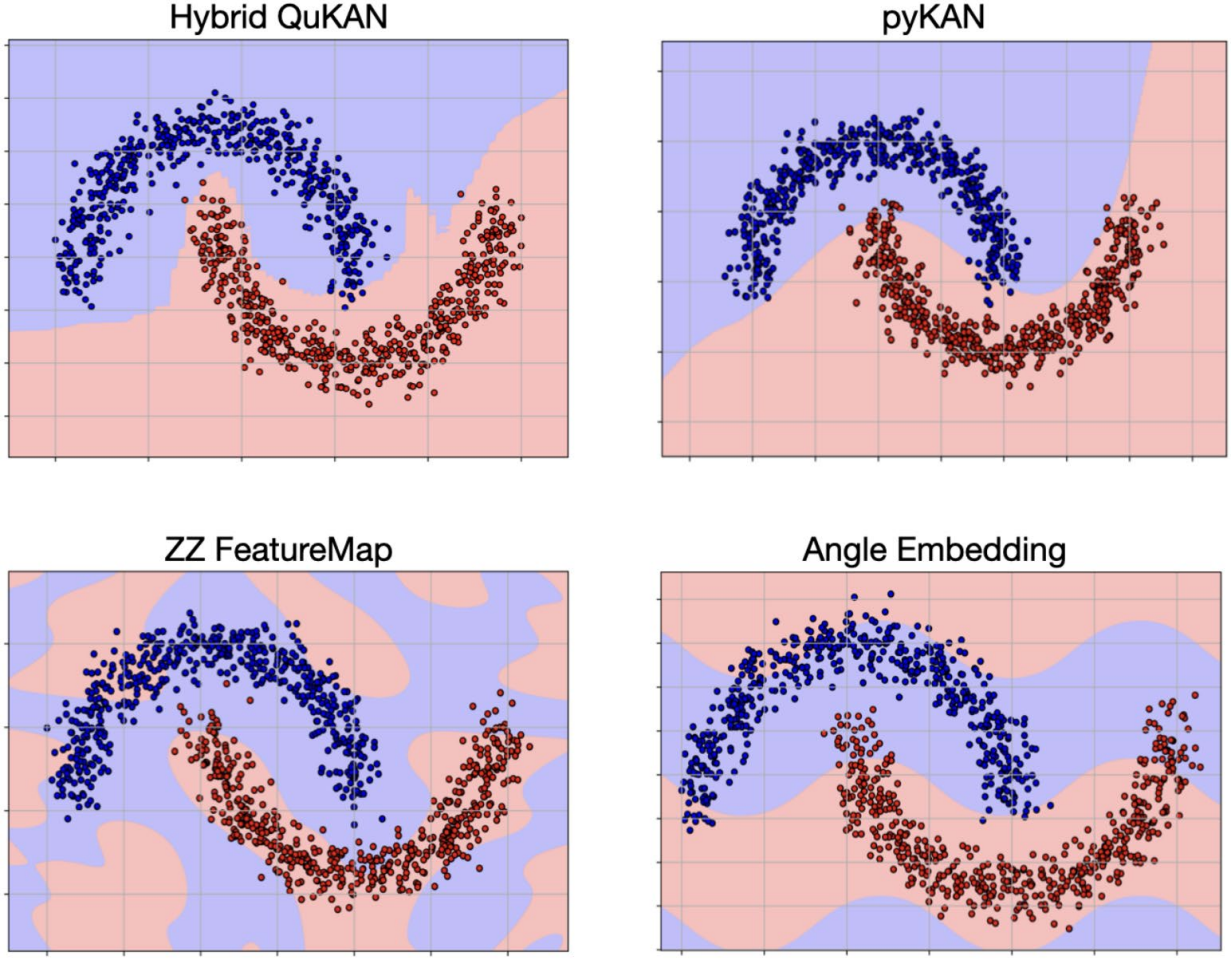
Decision boundaries on the moons dataset (with noise=0.1) for different models.

In order to compare the proposed Quantum KAN architecture to other quantum methods we chose binary classification as a first benchmark. We set up the network consisting of 2 layers and initialize each residual function as an equal superposition of 4 splines of degree 2. For the moons dataset we use a total of 1000 examples for training and the same amount of samples for testing. Both sets are independently sampled using a noise level of 0.1. To check the stability of the model as well as compare it to different quantum classifier models, we present in Fig. 6 the decision boundary after training our proposed QuKAN, together with that of other models.

Namely, we compare the performance and accuracy of our hybrid QuKANs with those of competing quantum models, as well as the classical pyKAN. For pyKAN we introduced a rigid grid and limited the maximum number of splines to 4 in order to ensure a fair comparison with the QuKAN architecture. For quantum methods, we mainly focus on Variational Quantum Classifiers^25,26^ that perform classification by processing the encoded data through a strongly entangling layer architecture^25^, followed by the evaluation of the expectation value of an observable on a single qubit. We also include different embedding strategies to enhance the performance of the quantum models, namely amplitude^20^ and angle embedding^27^. For amplitude embedding we also include ancilla qubits to enable a meaningful comparison, as the QuKAN implementation is essentially a Parametrized Quantum Circuit (PQC) that incorporates both pre-training and ancilla qubits. For the angle embedding^28^ we include a ZZ-feature map^28,29^. Finally, for the QKAN model proposed by Ivashkov et al^7,30^ we implemented a simple autograd based optimization algorithm of their proposed transfer function to be able to compare to our proposed QuKAN architecture. The classification performance can be seen in Table 1 while in Fig. 6 we present the predicted decision boundaries for the moons dataset for the 4 different models. It is easy to see that QuKAN and pyKAN predict similar boundaries, however the one predicted by pyKAN is smoother. Since the performance of full quantum models is highly sensitive to their embedding strategies^28,31,32^, this becomes a limiting factor in their effectiveness in the case of Angle Embedding, including ZZ feature maps, as well as Amplitude Embedding. Furthermore, we note that VQC approaches with trainable observables have demonstrated improved performance on the moons dataset^33^, however, we leave comparison to those methods as future work and focus on simpler model architectures that are closer in complexity to our proposed method.

**Table 1:** Comparison of the mean test accuracy for different machine learning models for the make Moons (with noise=0.1) and Iris dataset. All models are initialized with an comparable amount of parameters and trained for 20 epochs. For better comparison of QuKAN and pyKAN we scaled pyKAN down to a rigid grid and only two layers as well as 4 splines per residual function. For QKAN we used 2 hidden layers of width 3 alongside the input and output layers with Chebyshev polynomials^34^ up to degree of 3 for each. All models are evaluated over 4 different seeds and the mean test accuracy is presented. The Accuracies for different noise levels in the dataset can be found in the Supplementary Material.

Model	Mean test accuracy Moons	Mean test accuracy Iris
QuKAN	${\bf 97.94\%\pm 0.14\%}$	${\bf 100\%\pm 0\%}$
Rigid grid pyKAN	$97.74\%\pm 0.34\%$	$97.49\%\pm 0.014 \%$
MLP (2 layers)	$86.52\%\pm 0.93\%$	$81.14\%\pm 0.33\%$
MLP (4 layers)	${\bf 99.74\%\pm 0.15\%}$	${\bf 100\%\pm 0\%}$
VQC (amplitude embedding)	$84.78\%\pm 0.001\%$	$60.00\% \pm 0\%$
VQC (amplitude embedding + Ancillas)	$83.96\%\pm 0.001\%$	$60.00\% \pm 0\%$
VQC (angle embedding)	$80.18\%\pm 0.004\%$	$63.00\% \pm 0.04\%$
VQC (ZZ FeatureMap)	$81.21\%\pm 0.007\%$	$66.99\% \pm 0.05\%$
QKAN	$84.06\%\pm 0.005\%$	${\bf 100\%\pm 0\%}$

As presented in Table 1 the QuKAN and the rigid grid pyKAN show similar performance for both test sets. In the case of the moons dataset our QuKAN shows higher mean accuracies over different parameter initializations than the QKAN by Ivashkov et al.^7^, while their performance is similar for Iris. The QuKAN outperforms all the VQC methods with different embeddings for the tested datasets.

### Function Regression

**Fig. 7:**
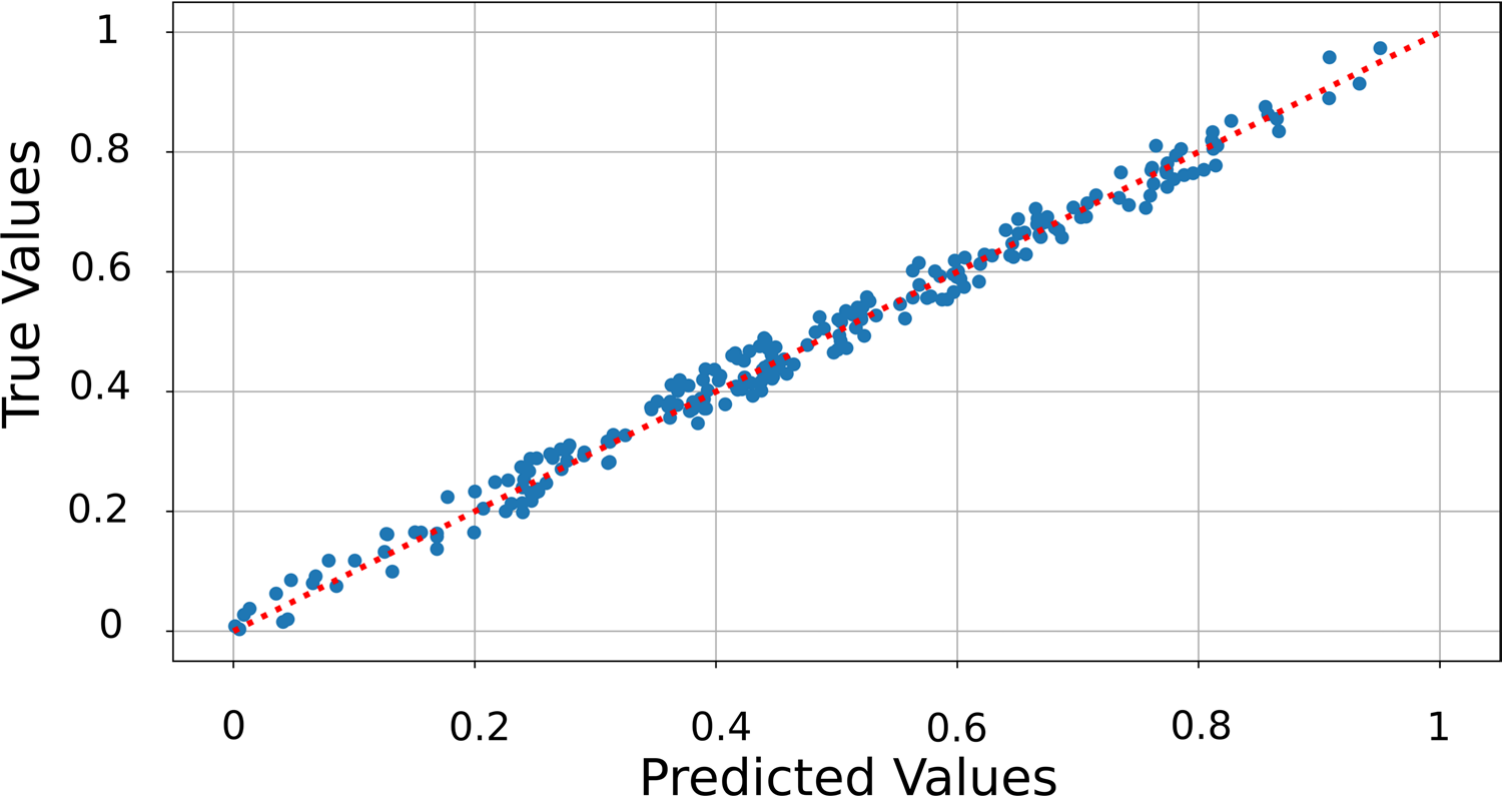
Comparison of the true against the predicted values for the regression task of the QuKAN. The red line indicates perfect prediction.

As demonstrated in the original generalization of the KAN by Liu et al^1^, KANs are particularly well-suited for function regression tasks due to their structured and interpretable composition of basis functions. In this section we want to show how the Quantum KAN can be used to fit a multivariate function. We choose

14 $$\begin{aligned} f(x_1,x_2) = 2x_1 -3x_2 +1 \end{aligned}$$

defined over the input domain $x_1,x_2\in [0,1]$. This function serves as a controlled benchmark to test the approximation capability of the model with limited number of parameters. We initialize the QuKAN model with two residual layers and limit it to 4 Splines basis function per residual. The result is visualized in Fig. 7.

To further evaluate the regression capabilities of our QuKAN model, we compare it to results reported in an implementation of a Quantum Kolmogorov Arnold Network proposed by Wakaura et al^10^. In this paper the sum of absolute distances between predicted and true function values is reported. We compare the models on the regression task of the function

15 $$\begin{aligned} f(x_0,x_1) = \ln \left( \frac{x_0}{x_1}\right) \end{aligned}$$

and optimize via Mean Squared Error loss. We start by calculating the Average, median, minimum and maximum of the sum of absolute distances of the predicted value to the true values for a batch size of 250 as shown in Table 2. For comparison to the model proposed by Wakaura et al, we set the train set to 10 samples and the test set to 50 samples. The results are summarized in Table 3. In this case we also include training on the proposed full QuKAN architecture.

**Table 2:** Average, median, minimum and maximum of the sum of absolute distances for the function regression for the hybrid QuKAN. We chose the training and test set to be of size 250.

Model	Sum abs. dist. avg.	Sum abs. dist. med.	Sum abs. dist. min.	Sum abs. dist. max.
QuKAN (2 layers)	0.7524	0.5451	0.0091	3.3094
QuKAN (1 layer)	0.8332	0.7157	0.0142	3.714

**Table 3:** Comparison of the average, median, minimum and maximum of the sum of absolute distances between the EVQKAN and (hybrid and fully) QuKAN for a function regression task. The values for the EVQKAN are taken from the Quantum KAN paper by Wakaura et al.^10^. Here the train set has 10 samples and the test set has 50 as in Wakaura et al.^10^.

Model	Sum abs. dist. avg.	Sum abs. dist. med.	Sum abs. dist. min.	Sum abs. dist. max.
QuKAN (2 layers)	0.6833	0.554	0.0015	3.3328
QuKAN (1 layer)	0.7437	0.5836	0.0008	3.7846
FQuKAN	0.995	0.7542	0.0079	3.6821
EVQKAN	1.229062	1.319659	0.753301	1.646876

### Pre-training has an effect

**Fig. 8:**
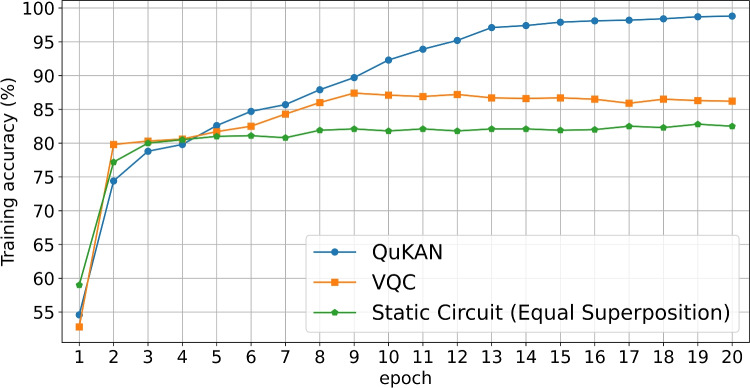
Comparison of the QuKAN training accuracy with a VQC architecture and a random number generator.

To analyse the effect of the pre-training of the QCBM encoded B-Spline basis functions, we perform the training of the binary classification of the moons dataset again. We compare the training behaviour of a VQC with a circuit of equal size as the residual functions to two versions of the QuKAN model: one with pre-trained embedded splines in the quantum residual functions, and one without pre-training initialized in an equal superposition state over the whole computational basis (Hadamard gates). While the output is still given by a projective measurement of the qubit position register, the architecture of the non pre-trained network is analogous to a Variational Quantum Classifier that includes ancillas. We compare the training accuracy over 20 epochs for all models over the moons dataset with noise level 0.1. The result is presented in Fig. 8.

Though both models seems to have an initial success in their learning, the accuracy of the QuKAN without pre-training quickly converges $87.6\%$, indicating that the model does rely on the splines encoded into the QCBM superposition state. We also investigate the effect of removing trainability of the residual function by removing parametrization on the quantum part and replacing by Hadamard gates applied on the complete computational basis. In this case the residual function is equal to a scalable SiLU function and a bias term.

### Summary of the Results

The presented benchmarks demonstrate that the hybrid Quantum Kolmogorov Arnold Network (QuKAN) is a viable implementation of the classical KAN. On binary classification such as the moons and the Iris datasets, QuKAN achieves high accuracies, outperforming Variational Quantum Classifiers with different embedding strategies. In comparison to classical methods such as MLP and KAN, it remains competitive in terms of interpretability over the number of parameters included for training. Additionally, we compared the classification performance to the QKAN proposed by Ivashkov et al^7^, where we added an autograd ADAM based learning algorithm to their transfer function.

For the function regression task, we could show that the QuKAN trains to fit a linear and a non-linear function. We also showed comparison to the EVKAN^10^ model. For the non-linear function we provided data indicating that the fully quantum architecture of the QuKAN residual function in the network is able to train on non linear regression tasks.

Finally, the ablation study on pre-training confirms its crucial role: models trained without the pre-trained QCBM-encoded spline basis quickly plateau during training. This emphasizes that the embedding into quantum residuals prior to training enhances the model’s learning capabilities.

## Discussion

In this work, we have demonstrated that the classical Kolmogorov Arnold Network as proposed by Liu et al^1^, can be effectively implemented into a quantum framework using a Quantum Circuit Born Machines approach. We encoded B-Spline basis functions into the amplitude structure of quantum states, allowing them to be interpreted probabilistically via projective measurements of the computational basis states. While we adopted B-Splines to remain consistent with the original KAN formulation, our method is not restricted to a specific choice of basis functions. In principle, any trainable basis set could be used.

By partitioning the qubit register into labelling and position subspaces, we enabled the encoding of multiple basis functions in quantum superposition, with tunable weighting across the function space. These weights are controlled by trainable parametrized unitaries, implemented via strongly entangling layers. This approach allows us to construct both a hybrid quantum KAN and a fully quantum residual analog, while preserving the compositional interpretability that distinguishes KAN from black-box neural networks^35,36^.

One of the main benefits of our quantum formulation, stemming from its close resemblance to the classical counterpart, is that it preserves interpretability as discussed in the original KAN by Liu et al.^1^. Moreover, by making use of quantum superposition, the model can represent an exponential number of functions while requiring only a linear growth in the number of qubits. Specifically, the ability to evaluate multiple basis functions simultaneously through projective measurements results in a compact model.

We validated the proposed architecture on toy classification and regression tasks, including moons and Iris datasets as well as multivariate function approximation. We compared the QuKAN to classical as well as quantum methods showing the mean test accuracy over different seeds, thereby showcasing its stability over parameter initialization. We found that the hybrid quantum KAN achieves performance comparable to classical methods, whereas the fully quantum KAN shows effectiveness only in the regression task. This limitation arises from the encoding scheme, where input vectors must be normalized to form valid quantum states, and superpositions must remain normalized throughout training. As a result, when optimization places high weight on the $\operatorname {SiLU}$ component, the spline coefficients are inevitably downscaled. In the residual formulation, this causes the basis function to dominate while the residual remains small, which can restrict expressivity. In contrast, HyQuKAN treats the $\operatorname {SiLU}$ term independently, providing a stronger foundation and greater flexibility.

Our results suggest that QuKAN provides a promising blueprint for the implementation of KANs into a quantum framework. Further research on more challenging benchmarks, combined with efforts to address current limitations, has the potential to unlock full capabilities of the model and expand its range of applications. As mentioned in the implementation details, the choice of having B-Splines as the pretraining of the model was due to its roots in the original KAN. In principle the choice of the initial functions is arbitrary when encoded in the proposed scheme. Further research can investigate the effect of choosing different pre-training function sets. Additionally, the network in its proposed form is static when it comes to depth and width. However, one of the major contributions of the original KAN was to make width and depth of the network arbitrary in contrast to the original KAR. The investigation of a “deep” version utilizing the proposed residual functions and a KAN like network structure could prove insightful. Finally, as shown in the Supplementary Material, limitations arise when summing over all splines in a residual function, however the choice of indices in the restriction of the sum is a hyperparameter. Further research can investigate the impact of different choices of this hyperparameter and how they align with the complexity of the dataset, especially in the FQuKAN architecture.

## Supplementary Information

Below is the link to the electronic supplementary material.

Supplementary Material 1

## Data Availability

Data and code will be made available on reasonable request. Correspondence and requests for materials should be addressed to M.K-E.
