# Supplementary material for: QuKAN: A Quantum Circuit Born Machine Approach to Quantum Kolmogorov Arnold Networks
Source: Sci Rep. 2025 Oct 9;15:35239. doi: 10.1038/s41598-025-22705-9 (PMC12511409; doi:10.1038/s41598-025-22705-9)
Supplement: Supplementary file 1 — Supplementary Material 1 [file 41598_2025_22705_MOESM1_ESM.pdf]

# Supplementary Material for “QuKAN: A Quantum Circuit Born Machine approach to Quantum Kolmogorov Arnold Networks”


**Yannick Werner[1,3,*], Akash Malemath[1,2], Mengxi Liu[3], Vitor Fortes Rey[3], Nikolaos Palaiodimopoulos[1,3], Paul Lukowicz[1,3], and Maximilian Kiefer-Emmanouilidis[1,2,3]**

[1]Department of Computer Science and Research Initiative QC-AI, RPTU Kaiserslautern-Landau, Kaiserslautern, Germany
[2]Department of Physics, RPTU Kaiserslautern-Landau, Kaiserslautern, Germany
[3]German Research Center for Artificial Intelligence (DFKI), Kaiserslautern, Germany
[*]mun60zor@rptu.de


## ABSTRACT


Kolmogorov Arnold Networks (KANs), built upon the Kolmogorov Arnold representation theorem (KAR), have demonstrated promising capabilities in expressing complex functions with fewer neurons. This is achieved by implementing learnable parameters on the edges instead of on the nodes, unlike traditional networks such as Multi-Layer Perceptrons (MLPs). However, KANs potential in quantum machine learning has not yet been well explored. In this work, we present an implementation of these KAN architectures in both hybrid and fully quantum forms using a Quantum Circuit Born Machine (QCBM). We adapt the KAN transfer using pre-trained residual functions, thereby exploiting the representational power of parametrized quantum circuits. In the hybrid model we combine classical KAN components with quantum subroutines, while the fully quantum version the entire architecture of the residual function is translated to a quantum model. We demonstrate the feasibility, interpretability and performance of the proposed Quantum KAN (QuKAN) architecture.


## Supplementary Material

### Limitations of Training When Summing Over All Splines

The initial state is given by:

$$|f_{\text{init}}\rangle = \sum_{i=0}^{N_L-1} c_i |i\rangle|\beta_i\rangle \tag{1}$$

and the evolved state is:

$$|f\rangle = (\hat{U}_L(\theta) \otimes \hat{I}_P)\,|f_{\text{init}}\rangle\,, \tag{2}$$

The projective measurement for a particular labelling state writes as follows:

$$p_j^\theta(x) = |\,\langle j,x|\,(\hat{U}_L(\theta) \otimes \hat{I}_P) \sum_{i=0}^{N_L-1} c_i\,|i,\beta_i\rangle\,|^2 = |\,\langle j|\,\hat{U}_L(\theta) \sum_{i=0}^{N_L-1} c_i\beta_i(x)\,|i\rangle\,|^2 = |\,\langle j|\,\hat{U}_L(\theta)\,|f(x)\rangle\,|^2$$

where in the last equation we just set for convenience $|f(x)\rangle = \sum_{i=0}^{N_L-1} c_i\beta_i(x)\,|i\rangle$. Now if we sum over all $j$ values we get:

$$p^\theta(x) = \sum_j p_j^\theta(x) = \sum_j |\,\langle j|\,\hat{U}_L(\theta)\,|f(x)\rangle\,|^2 = \sum_j \langle f(x)|\,\hat{U}_L^\dagger(\theta)\,|j\rangle\,\langle j|\,\hat{U}_L(\theta)\,|f(x)\rangle = \langle f(x)|f(x)\rangle \tag{3}$$

where we have used $\sum_j |j\rangle\,\langle j| = 1$ and the unitarity of the $\hat{U}_L(\theta)$. Here, we have to note that $|f(x)\rangle$ is generally not a normalized state. As a consequence, the final expression becomes independent of $\theta$, which prevents the model from being trainable. To retain the $\theta$-dependence, one may either restrict the summation to a subset of $j$ values or introduce parametrized gates in the position register.

## Tables

| Model | Noise level | | |
|---|---|---|---|
| | 0.2 | 0.3 | 0.5 |
| QuKAN | **93.48%** | **89.44%** | **83.68%** |
| Rigid grid pyKAN | 92.40% | 87.90% | 79.50% |
| VQC (Amplitude Embedding) | 82.60% | 82.10% | 70.10% |
| VQC (Amplitude Embedding + Ancillas) | 83.60% | 80.90% | 73.30% |
| VQC (ZZ FeatureMap) | 68.50% | 69.70% | 58.00% |
| VQC (Angle Embedding) | 79.70% | 79.80% | 53.10% |

**Table 1.** Test accuracy of various models trained for 20 epochs and for different noise levels on the moons dataset.

| Model | Optimizer | Learning Rate | Batch Size | Number of trainable parameters |
|---|---|---|---|---|
| QuKAN | ADAM | 0.1 | 45 | 24 |
| Rigid grid pyKAN | LBFGS | 0.1 | 1000 | 18 |
| MLP (2 layers) | ADAM | 0.1 | 45 | 43 |
| MLP (4 layers) | ADAM | 0.1 | 45 | 44 |
| VQC (Amplitude Embedding) | ADAM | 0.01 | 100 | 16 |
| VQC (Amplitude Embedding + Ancillas) | ADAM | 0.01 | 100 | 16 |
| VQC (Angle Embedding) | ADAM | 0.01 | 100 | 16 |
| VQC (ZZ FeatureMap) | ADAM | 0.01 | 100 | 16 |
| QKAN | ADAM | 0.1 | 100 | 84 |

**Table 2.** Optimization parameters for each model for the moons dataset.

| Model | Optimizer | Learning Rate | Batch Size | Number of trainable parameters |
|---|---|---|---|---|
| QuKAN | ADAM | 0.1 | 45 | 24 |
| Rigid grid pyKAN | LBFGS | 0.1 | 35 | 18 |
| MLP (2 layers) | ADAM | 0.01 | 60 | 43 |
| MLP (4 layers) | ADAM | 0.01 | 60 | 44 |
| VQC (Amplitude Embedding) | ADAM | 0.01 | 25 | 16 |
| VQC (Amplitude Embedding + Ancillas) | ADAM | 0.01 | 25 | 16 |
| VQC (Angle Embedding) | ADAM | 0.01 | 25 | 16 |
| VQC (ZZ FeatureMap) | ADAM | 0.01 | 25 | 16 |
| QKAN | ADAM | 0.1 | 20 | 84 |

**Table 3.** Optimization parameters for each model for the Iris dataset.

## Images

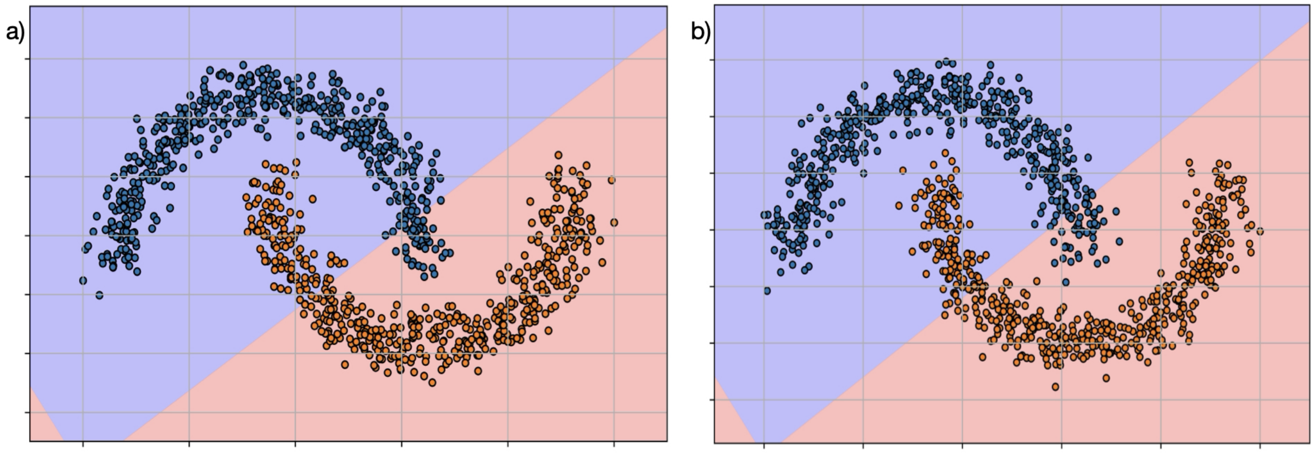


**Figure 1.** Decision boundaries of the Variational Quantum Classifier with different setups: a) Amplitude Embedding, b) Amplitude Embedding including a total of 4 ancillas. The dataset is the moons dataset with a noise of 0.1.

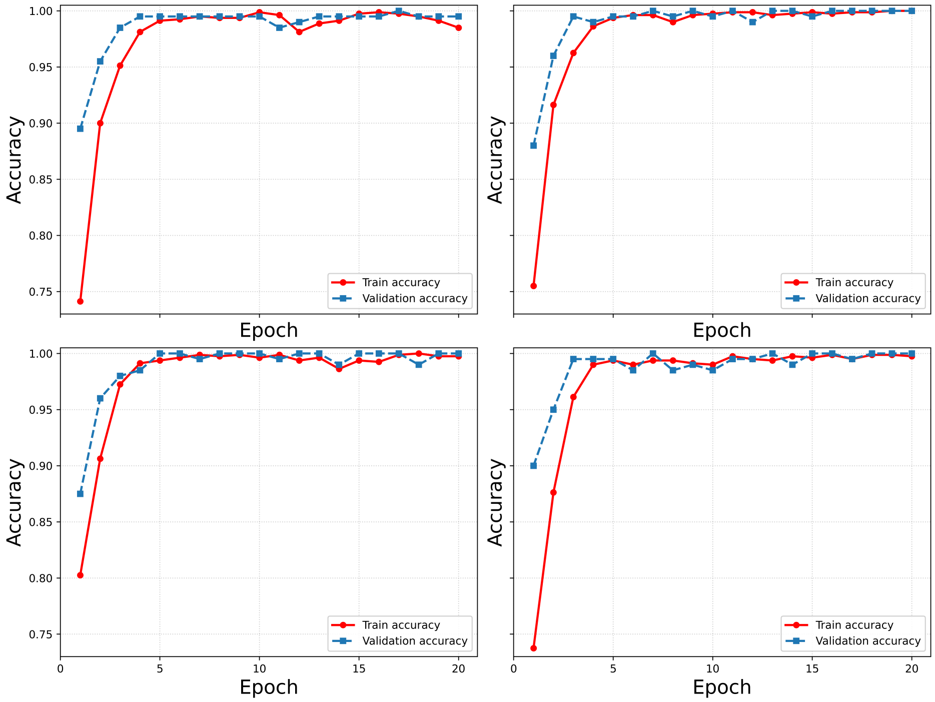


**Figure 2.** Training and validation accuracies over different seeds for the Moons dataset. It is visible that training as well as validation accuracy increase, indicating that the model is not overfitting. However, because the Moons dataset is relatively simple, models often achieve high accuracy without clear signs of overfitting.

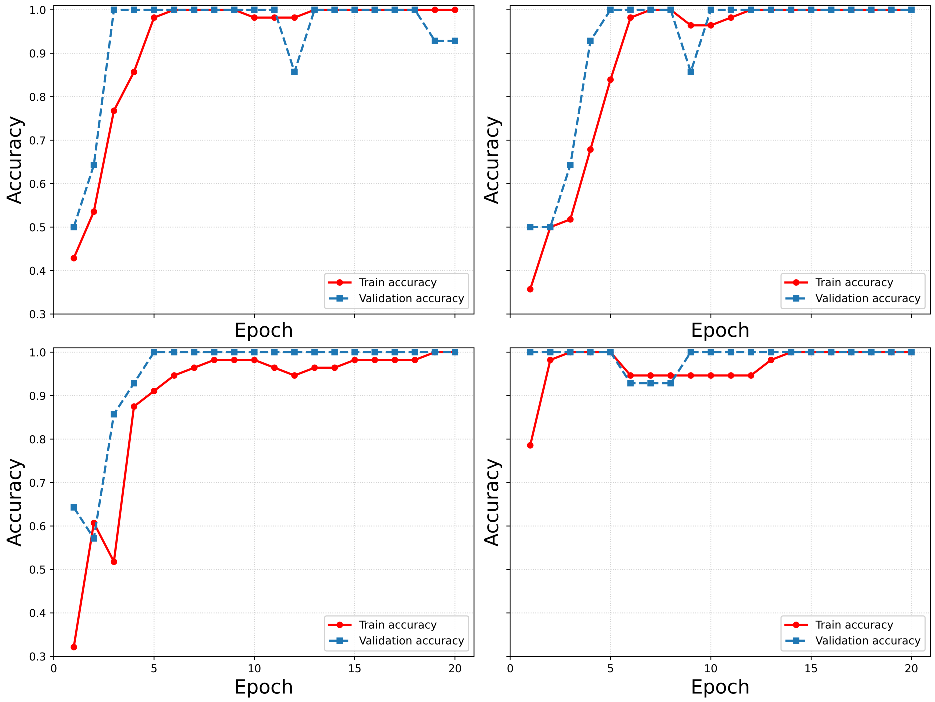


**Figure 3.** Training and validation accuracies over different seeds for the Iris dataset. It is visible that training as well as validation accuracy increase, indicating that the model is not overfitting. However, it should be noted that, because the Iris dataset is relatively simple, models often achieve high accuracy without clear signs of overfitting.
